# Improving the clinical trial landscape for Parkinson syndromes in Germany

**DOI:** 10.1007/s00702-026-03314-w

**Published:** 2026-10-24

**Authors:** Günter Höglinger, Karla Eggert, Joseph Classen, Thilo van Eimeren, Matthias Höllerhage, Wolfgang H. Jost, Jan Kassubek, Jochen Klucken, Thomas Koeglsperger, Rejko Krüger, Thomas Müller, Monika Pötter-Nerger, Peter Riederer, Alexander Storch, Jürgen Winkler, Ullrich Wüllner, Christoph Schindler, Paul Lingor, The German Parkinson’s Experts Meeting Group

**Affiliations:** 1 Department of Neurology, LMU University Hospital, Ludwig-Maximilians- Universität (LMU) München, Munich, Germany; 2 German Center for Neurodegenerative Diseases (DZNE), Munich, Germany; 3 Munich Cluster for Systems Neurology (SyNergy), Munich, Germany; 4 Department of Neurology, University Hospital Marburg, Marburg, Germany; 5 Dept. of Neurology, University Hospital Leipzig, Leipzig, Germany; 6 Dept. of Neurology, University Hospital Cologne, Cologne, Germany; 7 Dept. of Neurology, Hannover Medical School, Hannover, Germany; 8 Dept. of Neurology, Saarland University, Homburg, Germany; 9 Dept. of Neurology, University Hospital Ulm, Ulm, Germany; 10 Luxembourg Centre for Systems Biomedicine, University of Luxembourg, Esch-sur-Alzette, Luxembourg; 11 Centre Hospitalier de Luxembourg, Strassen, Luxembourg; 12 Luxembourg Institute of Health, Strassen, Luxembourg; 13 Alexianer St. Joseph-Krankenhaus Berlin-Weißensee, Berlin, Germany; 14 Dept. of Neurology, University Hospital Hamburg-Eppendorf, Hamburg, Germany; 15 Dept. of Psychiatry, Würzburg University, Würzburg, Germany; 16 Dept. of Neurology, University Medical Center Rostock, University of Rostock, Rostock, Germany; 17 German Center for Neurodegenerative Diseases (DZNE) Rostock- Greifswald, Rostock, Germany; 18 Department of Molecular Neurology, University Hospital Erlangen, Erlangen, Germany; 19 Center for Neurology, University Hospital Bonn, Bonn, Germany; 20 German Center for Neurodegenerative Diseases (DZNE), Bonn, Germany; 21 Zentrum für Klinische Studien and Early Clinical Trial Unit, Clinical Research Center, Medizinische Hochschule Hannover (MHH), Hannover, Germany; 22 Department of Neurology, TUM Hospital, Technische Universität München (TUM), Munich, Germany; 23 Department of Neurology, LMU University Hospital, Ludwig-Maximilians- University (LMU) Munich, Marchioninistr. 15, 81377 Munich, Germany

**Keywords:** Parkinson’s disease, Progressive supranuclear palsy, Multiple system atrophy, Corticobasal degeneration, Dementia with lewy bodies, Clinical trials, Infrastructures, Recruitment, German Parkinson study group

## Abstract

Germany has a longstanding tradition of excellence in neuroscience and Parkinson’s research, supported by outstanding academic institutions, specialized clinical centers, and a strong scientific community. Building on this strong foundation, the country now has the opportunity to transform its clinical research landscape into a more integrated ecosystem for Parkinson syndromes. Despite major advances in molecular understanding, biomarker development, and therapeutic innovation, important structural, regulatory, financial, and organizational barriers continue to limit the full translational potential of German Parkinson research. Participants in an expert workshop identified identify recurring challenges, including fragmented trial infrastructures, slow and bureaucratic regulatory procedures, insufficient funding for investigator-initiated clinical research, limited integration of patient organizations, and suboptimal patient recruitment pathways. This workshop-derived perspective outlines a strategic framework to elevate Germany from a country with excellent individual centers to a globally competitive and integrated hub for innovative Parkinson clinical research. Key recommendations include transforming the German Parkinson Study Group into a nationally coordinated, service-oriented trial network; establishing centralized digital and regulatory infrastructures; diversifying public and philanthropic funding; implementing platform and observational trial structures; strengthening incentives for participating centers; and enhancing patient engagement through national registries, decentralized trial approaches, real-world data based evidence surveillance, and systematic integration of patient organizations. The authors argue that Germany must move beyond isolated university-based structures toward a nationally coordinated and highly efficient research ecosystem capable of rapidly delivering disease-modifying and precision medicine trials. Such a transformation could strengthen Germany’s international position in neurodegenerative disease research and accelerate therapeutic innovation for patients with Parkinson syndromes.

## Introduction

Over the past decades, research into Parkinson syndromes, i.e., Parkinson’s disease (PD) and atypical Parkinson syndromes, including Multiple System Atrophy (MSA), Dementia with Lewy Bodies (DLB), Progressive Supranuclear Palsy (PSP) and Corticobasal Degeneration (CBD), has achieved transformative scientific, diagnostic and therapeutic advances. These include the development and refinement of dopamine replacement therapies (Riederer et al. 2025; Trenkwalder et al. 2011; Poewe et al. 2007), the implementation of deep-brain stimulation and infusion-based therapies (Deuschl et al. 2006; Schuepbach et al. 2013), the elucidation of genetic and environmental contributors to disease risk (Zimprich et al. 2004; Lill et al. 2012; Nalls et al. 2014), the recognition of alpha-synuclein pathology (Spillantini et al. 1997; Braak et al. 2003), the characterization of disease progression from prodromal manifestations to motor and cognitive dysfunction (Berg et al. 2015; Postuma et al. 2015) and the current transformation towards a biology-based disease definition (Höglinger et al. 2024; Simuni et al. 2024), biomarker-based diagnosis (Mollenhauer et al. 2011, 2019; Bernhardt et al. 2025) and digital assessments (Mikolaizak et al. 2022; Brzenczek et al. 2024; Koeglsperger et al., 2026). The field of atypical Parkinson syndromes, including progressive supranuclear palsy, corticobasal degeneration, and multiple system atrophy, has seen major advances recently with neuropathologically based disease definitions, evidence-based clinical diagnostic criteria, and increasingly objective imaging and fluid-biomarker-based diagnosis and exciting advances in digital outcomes and innovative therapeutic trials (Brendel et al. 2020; Höglinger et al. 2017; Palleis et al. 2026; Stamelou et al. 2021; van Eimeren et al. 2019; Wenning et al. 2022).

The field is now entering a new era driven by advances in molecular neuropathology, biomarker discovery, proteomics and (epi-)genomics, multimodal imaging, digital medicine, artificial intelligence, and precision therapeutics. Moreover, therapeutic advances in other neurodegenerative disorders, such as the introduction of anti-amyloid antibody treatments for Alzheimer’s disease and antisense oligonucleotide-based therapies in motoneuron diseases, lay out a roadmap for the future therapeutic developments in Parkinson syndromes. These developments are reshaping clinical trial methodology and increase the need for adaptive, biomarker-driven, and platform-based study designs.

Despite strong scientific tradition and methodological excellence in the field of Parkinson syndromes, ample chances remain for the international research community for improvements in conducting rapid, efficient, and innovative clinical trials. Expert discussions among German clinician-scientists in November 2025 in Frankfurt/Main identified potential structural and socio-cultural barriers that may compromise Germany’s competitiveness in the international trial ecosystem. These include fragmented infrastructures, prolonged regulatory processes, inadequate institutional incentives and insufficient funding mechanisms, limited patient engagement, and the absence of a nationally coordinated trial platform.

This workshop-derived perspective synthesizes the key strategic themes emerging from these discussions and proposes a roadmap to improve the clinical trial research landscape for Parkinson syndromes, with a specific focus on the current situation and opportunities in Germany.

### Workshop process and scope

The workshop took place during the German Parkinson’s Experts Meeting in Frankfurt/Main in November 2025. An introductory presentation on past achievements and current challenges was followed by an open discussion of structural barriers and possible solutions. The discussion was recorded and transcribed, structured into an initial manuscript and circulated twice to all authors for comments and proposed revisions. The lead author incorporated these contributions, and all authors agreed to submission. All listed authors contributed actively to the workshop and manuscript development. This article documents an expert perspective rather than a systematic or narrative literature review. No formal consensus procedure or quantitative assessment of agreement was undertaken; approval of the manuscript does not imply quantified agreement on each recommendation.

### Structural challenges in the German clinical trial landscape

One of the most prominent themes emerging from the expert discussions was the perception that the German Parkinson Study Group (GPG) has not yet unfolded its full potential to meet the requirements of modern clinical trial research. The current structures appear not optimally adapted to the increasing complexity of biomarker-driven, multi-center, and internationally coordinated studies, which often require genetic analyses, fluid biomaterial and tissue collection and processing, longitudinal multimodal MRI and PET-imaging, digital video- and sensor-based analyses, a highly qualified staff comprising study coordinators, nurses, and physicians in sufficient number and qualification to allow independent teams of investigators and raters.

Although the country hosts numerous highly qualified academic and non-academic movement disorder centers, these institutions frequently operate under high economical demands and in relative isolation. This pressure to optimize processes and staff allocations as well as geographical fragmentation limits the flexibility to meet sponsors’ demands, complicates standardization, and slows recruitment. In contrast, other countries have developed nationally coordinated infrastructures capable of rapidly implementing multi-center studies, including adaptive platform trials and multi-arm multi-stage trial concepts. German experts emphasized that future success will increasingly depend less on isolated excellence at individual institutions but rather on the establishment of flexible and integrated national ecosystems for clinical research.

Regulatory complexity represents another important challenge. Participants described a dramatic increase in bureaucratic requirements over recent decades, with ethics submissions and regulatory interactions becoming increasingly time-consuming and resource-intensive. Processes that previously required minimal documentation now involve extensive submissions to ethics committees, and data protection and regulatory authorities, resulting in substantial delays and cost. These burdens disproportionately affect investigator-initiated trials and smaller academic centers and may affect Germany’s attractiveness for both investigator-initiated and industry-sponsored studies. These concerns are consistent with policy recommendations on clinical trial infrastructure and financing and a joint academia–industry workshop involving the KKS network (Wissenschaftsrat 2018; Peters et al. 2023).

For objective context, the vfa’s ClinicalTrials.gov analysis identified 519 industry-funded interventional drug studies starting in 2023 with German participation, compared with 641 in 2016 and 524 in 2022. In the 2023 comparison, Germany was behind the United States, China and Spain and narrowly ahead of the United Kingdom and Canada (Verband et al. 2023). The analysis covered all indications and phases from early phase 1 to phase 4; it does not establish relative Parkinson-specific performance. Counts are not population-adjusted and do not measure recruitment, trial quality or start-up time; multinational studies may be counted in several countries.

Insufficient and unstable funding structures further impair the sustainability of clinical trial infrastructures. While Germany invests heavily in basic science and large-scale institutional research programs, funding dedicated to operational clinical trial infrastructure has remained fragmented and temporary. Study nurses, coordinators, and regulatory personnel are often financed through project-specific mechanisms that provide little or no continuity. Structured formation programs are rare and not standardized. This lack of structural support undermines long-term planning and contributes to workforce instability.

The discussions also highlighted opportunities to strengthen philanthropic support for clinical research in Germany. Participants considered, private donors, charitable foundations, and family offices potential complementary sources of support. Several experts emphasized that philanthropic funding has become an essential pillar of successful neurodegenerative research infrastructures in other countries and raised the question how to encourage greater involvement from private sponsors in Germany to the benefit of the society’s functionality.

Patient recruitment represents a major bottleneck in the clinical trial landscape. Germany’s healthcare system is among the most advanced and comprehensive worldwide, providing broad access to high-quality diagnostic and therapeutic care largely independent of socioeconomic status. All insured individuals generally have access to the full spectrum of approved symptomatic treatments for Parkinson syndromes, irrespective of insurance type. While this represents a major societal achievement, it may paradoxically reduce motivation for clinical trial participation.

Because patients already receive excellent routine care, the perceived individual benefit of enrolling in a clinical trial may appear limited, whereas the associated burden—including additional visits, travel, study procedures, uncertainty regarding treatment allocation, and potential side effects—may weigh more heavily in the personal risk-benefit assessment. Trials investigating disease-modifying therapies with uncertain short-term benefits may therefore appear less attractive in a disease area with numerous established symptomatic treatment options.

In contrast, recruitment for rapidly progressive neurodegenerative disorders without effective standard therapies, such as atypical Parkinson syndromes or amyotrophic lateral sclerosis, may be motivated by the perception of a far greater unmet therapeutic need and a stronger urgency to participate in experimental research. However, rarity, diagnostic uncertainty, disability and caregiver burden can also constrain recruitment in atypical Parkinson syndromes.

An additional bottleneck is the limited integration of office-based neurologists into clinical trial pathways. Since most people with Parkinson syndromes receive long-term care in the outpatient setting, community neurologists and regional referral networks will be essential for identifying suitable participants—particularly for future biomarker-defined and disease-modifying trials. Stronger links between specialized trial centres and ambulatory care, for example through structured referral networks such as ParkLink (www.parklink.info), could substantially improve trial readiness and recruitment efficiency.

The discussions further highlighted cultural and societal factors that may additionally compromise recruitment in Germany. Participants called for stronger partnerships with some patient organizations to support informed research participation; the workshop did not assess individual organizations or establish a national ranking of patient advocacy advocacy. In the participants’ experience, public awareness of research participation could be strengthened, and clinical trial involvement may not always be perceived as a collective societal contribution to therapeutic innovation. Consequently, both patients and healthcare professionals may face barriers to engagement in research activities, while patients frequently lack the community support and encouragement needed to participate in demanding longitudinal studies.

### Transforming the German Parkinson study group into a national infrastructure

A central strategic recommendation emerging from the discussions is the modernization and repositioning of the GPG. Rather than functioning as a historically evolved academic consortium, the GPG should develop into a modern, service-oriented national competence network for clinical trials.

Participants repeatedly stressed that future structures should not depend on individual personalities or dominant institutions but instead operate as scalable and neutral infrastructures capable of serving the entire national Parkinson research community. Such a transformation would require professionalized management structures, centralized operational support, harmonized quality standards, and broad integration of academic and non-academic study sites.

A major component of this modernization process would be the creation of a centralized digital platform. Experts emphasized that Germany urgently requires a national interface for patients, sponsors, and investigators, similar to “trial finder” systems successfully implemented in the United States. Such a platform could provide real-time information on recruiting studies, facilitate patient matching, improve communication between centers and sponsors, and establish transparency regarding study capabilities and timelines.

Such a platform should also be designed to interface with emerging real-world health data infrastructures and electronic health records. In Germany, the secondary use of health data is outlined by the Health Data Use Act (GDNG) and the Digital Act (DigiG) improving the usability of health data derived from every day healthcare procedures. These acts are part of Germany’s healthcare digitalisation strategy, marking a significant step in the country’s efforts to connect its healthcare system to the European Health Data Space (EHDS). These initiatives for the secondary use of health data create an opportunity to complement prospective trial data with routinely collected clinical information. In the longer term, this linkage could support feasibility assessments, patient identification, external control concepts, adaptive and in-silico trial designs, and the generation of real-world evidence on specific interventions and standards of care.

Establishment of regional referral networks connecting office-based neurologists with specialized trial centres should become a core function of the modernized GPG. Beyond facilitating recruitment, such networks would enable systematic identification of patients eligible for biomarker-driven and disease-modifying trials, thereby strengthening national trial readiness.

Beyond improving recruitment, a centralized digital infrastructure would substantially increase the visibility of German clinical trial activities. Pharmaceutical companies increasingly seek efficient and predictable collaboration networks with harmonized operational procedures and transparent performance metrics. A nationally coordinated infrastructure could therefore significantly improve Germany’s attractiveness for industry-sponsored studies.

## Funding, sustainability, and professionalization

The expert discussions made clear that sustainable clinical trial infrastructures cannot rely solely on temporary project funding or individual academic engagement. A future-oriented Parkinson trial ecosystem in Germany will require long-term structural financing mechanisms that combine public funding, institutional commitment, and philanthropic support.

Participants emphasized the need for targeted funding programs from German and European agencies specifically dedicated to clinical trial infrastructure in addition to basic science. Such funding could maintain the dedicated Clinical Trial Units for neurodegenerative diseases and the large observational cohorts already established, requiring sustainable operational support at the German Center for Neurodegenerative Diseases (DZNE) and the German Parkinson’s and Movement Disorders Society (DPG). A tight collaboration with existing complementary structures at the DZNE sites, the DPG-associated university hospitals, and Germany’s specialized Parkinson’s hospitals would further strengthen the translational power of such a clinical network.

At the same time, philanthropic funding was repeatedly identified as a major untapped opportunity. Experts noted that countries such as the United States and the United Kingdom have successfully integrated patient organizations, private donors, and charitable foundations into the financing of clinical trial ecosystems. Participants saw opportunities to expand such partnerships in Germany; selected organizational examples do not establish national differences in philanthropic culture. Several discussants argued that future efforts should more actively engage family offices, foundations, and major private donors in order to establish sustainable support structures for personnel, digital infrastructure and young investigator programs.

Illustrative funding examples include Parkinson’s UK, which reported more than £130 million invested in Parkinson research since 1969 in February 2026 (Parkinson’s 2026), and the German Parkinson Stiftung, which allocated €2.3 million to six Leuchtturm projects over three years starting in 2025 (Parkinson Stiftung, 2025). These figures describe a cumulative investment over decades and a specific funding programme, respectively. They are not comparable national totals or annual budgets and are not restricted to clinical trials, but illustrate opportunities for complementary charitable research support.

The discussions also highlighted the need for realistic economic frameworks for participating centers. Current reimbursement structures frequently fail to reflect the true complexity and administrative burden of clinical trial work. Without adequate compensation for infrastructure, administrative support, and personnel costs, hospitals face increasing difficulty in justifying research activity within economically constrained healthcare systems. The establishment of transparent national costing standards could strengthen negotiating positions with sponsors and improve institutional willingness to participate in clinical research. The need for transparent costing and the differing perspectives of academic sponsors and industry on appropriate financing have also been discussed by Peters et al. (2023).

### Reducing bureaucratic complexity through centralized support

The increasing regulatory complexity of clinical research was identified as one of the most important obstacles to international competitiveness. Experts repeatedly emphasized that Germany requires mechanisms to reduce duplication of effort and to support study centers in navigating regulatory processes more efficiently.

A centralized regulatory helpdesk embedded within a strengthened national Parkinson trial network could substantially improve efficiency. Such a structure could support ethics submissions, harmonize legal templates, coordinate interactions with federal authorities, and provide standardized operating procedures across participating centers. Shared expertise would be particularly valuable for smaller and non-university centers that currently lack dedicated regulatory personnel.

Importantly, the discussions also emphasized the need for stronger collaboration between experienced university centers and smaller institutions. Larger centers already possess substantial expertise in regulatory processes, contract negotiations, and operational trial management. A national infrastructure could help distribute this expertise more effectively and thereby improve the performance of the entire network.

### Platform trials, observational cohorts, and precision medicine

Several experts identified platform trials and large observational cohorts as strategic opportunities for enhancing the German Parkinson trial ecosystem. Adaptive platform structures offer considerable advantages, including shared infrastructure, improved efficiency, simultaneous evaluation of multiple therapies, and closer integration with biomarker-driven precision medicine approaches.

Shared infrastructure should accommodate disease-specific requirements: PD, MSA, PSP and CBD differ in diagnostic certainty, progression, recruitment and suitable outcomes. Platform and cohort designs therefore require separate scientific justification for each disease rather than treating Parkinson syndromes as a homogeneous trial population.

At the same time, observational cohorts may represent a realistic and achievable entry point for infrastructure development. Germany already possesses internationally recognized expertise in imaging, biomarker, and atypical Parkinson syndrome cohorts, including the German Imaging Initiative for Tauopathies (GII4T), the DescribePSP and ProPSP cohorts, which are currently being opened up for additional study cohorts inclunding MSA, CBD and prodromal and motor-manifest PD (Brendel et al. 2020; Bischof et al., 2024; Respondek et al., 2021; Saw et al. 2026). These infrastructures could serve as foundational elements for future interventional platform studies and precision medicine initiatives.

Beyond their role as observational resources, longitudinal deeply phenotyped cohorts are increasingly considered as important infrastructures providing trial-readiness. Existing cohorts with standardized clinical phenotyping, multimodal imaging, fluid biomarkers and genetic characterization provide an efficient recruitment pool for innovative precision medicine trials. Such infrastructures will facilitate rapid identification of suitable participants for clinically and/or biologically defined subgroups, including patients with GBA1 variants, α-synuclein seed amplification assay positivity, or individuals in early or de novo disease stages, thereby potentially improving recruitment, trial readiness and feasibility.

**Fig. 1:**
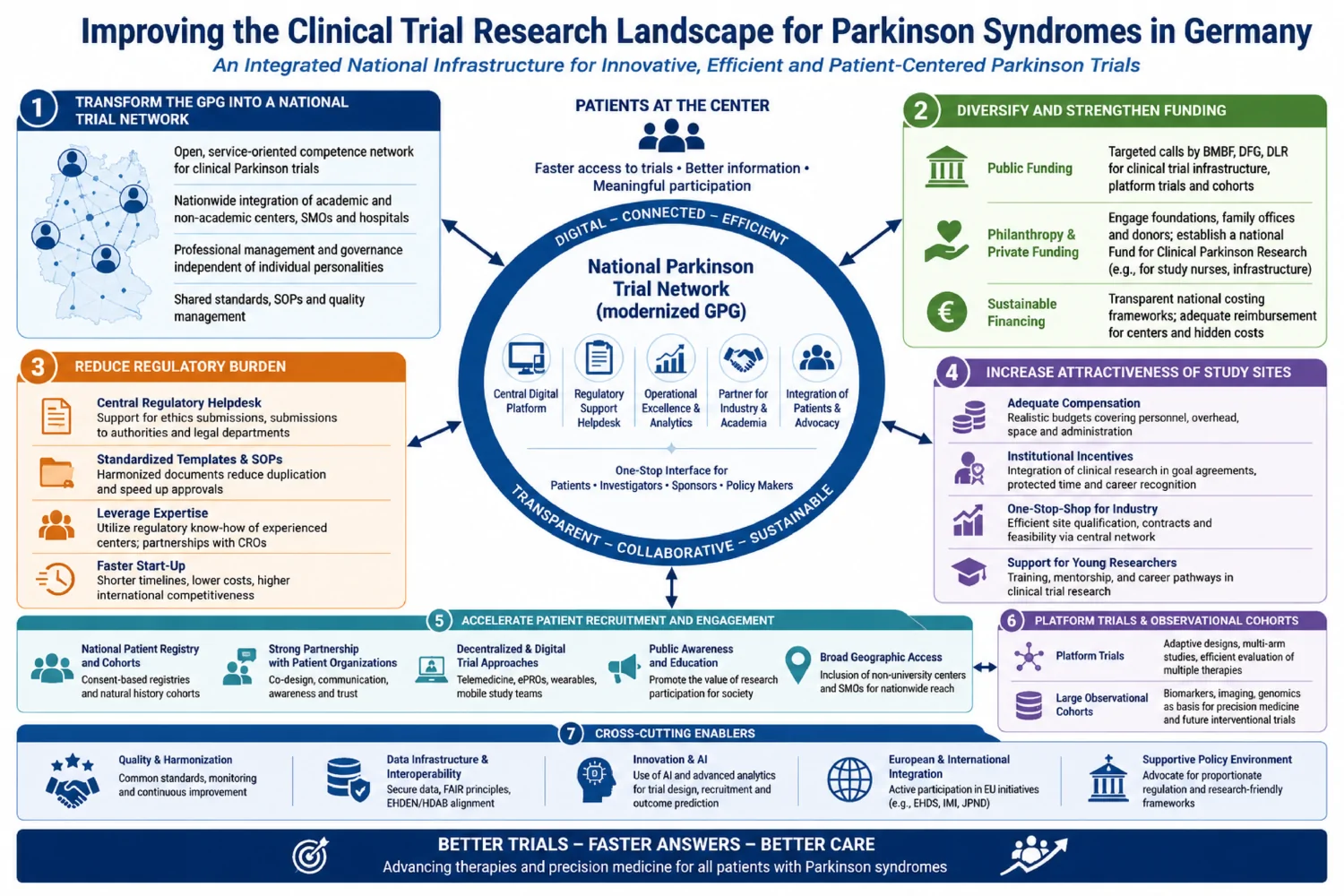
A concept to improve the clinical trial landscape for Parkinson syndromes in Germany (generated with ChatGPT based on the authors’ intellectual input

Importantly, such cohorts also create valuable academic opportunities for younger investigators through biomarker analyses, imaging studies, and translational research projects. Participants repeatedly stressed that sustainable clinical research ecosystems must provide meaningful academic perspectives for early-career researchers if long-term engagement is to be maintained (Fig.1).

### Patient engagement and the cultural dimension of research participation

The discussions highlighted that engaging patients and improving recruitment is not only an organizational challenge but has also socio-economic and cultural aspects (Smith et al. 2025; Fomo et al. 2025). Participants considered stronger public engagement a priority; the workshop did not establish international differences in civic engagement or a causal relationship between welfare provision and research participation.

Experts repeatedly emphasized the importance of stronger collaboration with patient advocacy organizations, public awareness campaigns, and broader societal recognition of research participation. National patient registries, decentralized trial approaches, telemedicine solutions, and mobile study infrastructures may help reduce barriers to participation and improve accessibility.

Patient involvement should extend beyond recruitment to priority setting, protocol design, assessment of participation burden, outcome selection, governance and dissemination. These roles should be developed with people living with Parkinson syndromes and caregivers, using accessible formats and support for sustained participation.

At the same time, patient motivation may increase when trial participation is framed not only as an individual therapeutic opportunity but also as a meaningful societal contribution. Several discussants argued that a stronger public narrative emphasizing solidarity, scientific progress, and collective responsibility could improve both recruitment and public support for clinical research.

### Existing national german infrastructures for clinical research

Several national infrastructures to support clinical trials in PD and other neurodegenerative disorders already exist in Germany:

#### The German competence network for Parkinson syndromes

 (Kompetenznetz Parkinson, KNP, www.kompetenznetz-parkinson.de) is a nationwide collaborative research network founded in 1999, funded by the German Federal Ministry of Research, to unite clinicians, basic scientists, and clinical researchers working on Parkinson’s disease. Its mission is to accelerate progress in understanding disease mechanisms, improve diagnostic and therapeutic strategies, and translate scientific discoveries into clinical practice with the ultimate goal of improving patient care and enabling earlier diagnosis, disease prevention, and disease-modifying treatments. The KNP integrates university hospitals, specialized Parkinson centers, rehabilitation facilities, and research institutions across Germany into a coordinated infrastructure for clinical and translational research. The network supports multicenter studies, standardized clinical data collection, biobanking, and collaborative research while promoting education, quality assurance, and rapid knowledge exchange between researchers and healthcare professionals. An essential pillar of the organization is the GPS, which coordinates national multicenter clinical studies.

####  The German society for Parkinson and movement disorders

(DPG, www.parkinson-gesellschaft.de), founded in 1984, is dedicated to advancing research on Parkinson’s disease and improving patient care. The society brings together neurologists, basic scientists, and other healthcare and research professionals with expertise in Parkinson’s disease and movement disorders, recognizing interdisciplinary collaboration as a key driver of progress in diagnosis and treatment. Through its support of scientific research, education, and early-career investigators, the DPG aims to improve clinical care while advancing early diagnosis, disease prevention, and ultimately the development of curative therapies for Parkinson’s disease and related disorders. The society also promotes scientific exchange by organizing the biennial German Congress for Parkinson and Movement Disorders.

#### The German center for neurodegenerative diseases

(DZNE, www.dzne.de) operates a nationwide research network with 10 sites that integrates basic science, population studies, and clinical research to advance the understanding and treatment of neurodegenerative disorders. To support high-quality clinical research, the DZNE provides standardized infrastructures for protocol development, regulatory compliance, patient recruitment, biomaterial handling, and data management, ensuring harmonized procedures and reproducible study outcomes across sites. In 2023, the Helmholtz Association approved funding of 35 million euros to establish Clinical Trial Units (CTUs) within the DZNE network – located in Bonn, Dresden, Magdeburg, Munich, Rostock, and Tübingen – as central facilities dedicated to conducting multicenter clinical studies in neurodegenreative disorders with unified methodological and operational standards.

####  The network of coordination centers for clinical trials

(KKS, www.kks-netzwerk.de) is the German network of academic clinical trial coordination centers, providing methodological, regulatory and operational expertise for the planning, conduct, quality assurance and analysis of clinical studies in University Medicine. It originated in 1999, when the German Federal Government funded the establishment of Coordinating Centres for Clinical Studies at German Medical Faculties.

#### The network of university medicine

(NUM, www.netzwerk-universitaetsmedizin.de/en) was established in April 2020 as a national response to the COVID-19 pandemic, with the aim of coordinating clinical COVID-19 research across German university medicine. It rapidly brought together all 37 German academic university medical centres and initiated multicentre research projects, shared data structures and research infrastructures. Over time, NUM evolved from a crisis-response mechanism into a broader platform for cooperative, cross-site clinical research, data use, biosampling and study readiness in university medicine. Its current trajectory is toward long-term institutionalisation as a national research infrastructure, with permanent funding agreed in 2026. Specialized networks have been established within the NUM. Currently, three specialized networks are listed within the NUM: (1) Infectious Diseases Research Network/SNID (2) Critical Care Medicine Research Network/SN CritCare, and (3) Stroke Research Network/SN Stroke. SNID was the first specialized network; the critical care medicine and stroke networks were launched in February 2026. Both national academic research networks KKS-network and NUM have begun to collaborate more closely. Whereas NUM is the national platform for coordinated university-medicine research in Germany, the KKS-Network is the specialist infrastructure for GCP-compliant clinical trial conduct. NUM connects the system, KKS operationalizes studies.

As far as funding is concerned, the KNP and DPG are financed primarily through membership dues and private donations. The funding allocated to the DZNE‑CTU network is primarily intended to establish the underlying infrastructure, whereas the operational costs required for running clinical studies must be covered through regular DZNE resources or external third‑party funding. The NUM is established and maintained by the federal government as a national research infrastructure. NUM provides project-based funding for the development and operation of shared national infrastructures—and is currently shifting from a model based on short-term projects toward a permanently funded research infrastructure. The KKS Network, on the other hand, is primarily funded as a membership and expertise network through membership fees, project grants, and training programs.

The proposed network would provide disease-focused coordination across these complementary resources: cohort and biomarker expertise at DZNE, methodological and operational trial support at KKS/ZKS centres, and cross-site research coordination through NUM. Its added value should be assessed through agreed referral, feasibility and service arrangements, avoiding duplication of existing capabilities.

### The european perspective

Many participants emphasized that Germany’s future performance in clinical trials cannot be considered independently of broader European developments. Legal frameworks such as the European Health Data Space and multinational neurodegenerative disease platforms may provide important mechanisms to funnel Germany into harmonization and international integration (Raab et al. 2023; Giehl et al. 2024).

KKS network and NUM have already been highlighted as existing networks for clinical research. The KKS Network hosts Germany’s connection to ECRIN, the European Clinical Research Infrastructure Network, whereas NUM is currently developing its European role more through disease-, data- and preparedness-oriented research infrastructures.

Certainly challenging, a German initiative has to actively position itself in the emerging European research infrastructures. A modernized national Parkinson trial network could play an important role to position Germany as a more attractive partner in future multinational precision medicine initiatives, alongside strong research communities and infrastructures in other countries within the European and global context.

## Conclusion

Germany possesses exceptional scientific expertise in Parkinson syndromes, outstanding clinical research facilities, highly qualified and motivated clinician scientists and the largest European population covered by a homogeneous healthcare framework. Participants identified structural and socio-economic barriers that may limit the translation of these strengths into efficient clinical trial delivery.

Improving the clinical trial landscape requires a coordinated, multi-level transformation. The most immediate priorities are the modernization of the GPG into a nationally coordinated trial network, implementation of a national Parkinson Trial Finder supported by regional referral networks, establishment of centralized regulatory support, and sustainable funding for trial infrastructure. Additional measures — Including platform trials, longitudinal cohorts, and stronger patient engagement — could further strengthen Germany’s long-term competitiveness.

The development of the GPG into a digitally integrated, nationally visible, service-oriented trial network generating real-world data-driven evidence represents a central strategic opportunity. Such a transformation could establish Germany as a leading European hub for innovative clinical Parkinson research and accelerate the development of urgently needed disease-modifying therapies.

Given Germany’s existing national infrastructure for clinical research, it seems promising to integrate the GPG in collaboration with DZNE, DPG, KKS and NUM as a new “Specialized Network for Parkinson’s and Neurodegenerative Diseases”. Within such a network, pharmaceutical research is being conducted that is increasingly highly regulated and, in some cases, extremely complex — research requiring complementary scientific, regulatory and operational expertise, with responsibilities agreed among the participating organizations. The stringent regulatory and operational requirements could be met by establishing a long-term partnership between this Parkinson’s network and an established clinical trial centre.

Implementation would require agreement on scientific priorities, patient representation, site access, sponsor responsibilities, accountability and conflicts of interest. Financing should distinguish continuing coordination and personnel costs from study-specific expenditure. The workshop did not establish a legal structure, an approved governance model or secured funding. Initial steps could map existing services and test agreed functions in pilot studies, measuring site activation, contract turnaround, recruitment and retention using consistent definitions.

Looking beyond the optimization of current clinical trial infrastructures, future ecosystems for Parkinson research should increasingly integrate longitudinal patient cohorts, biomarker platforms and adaptive platform trials into a seamless translational framework. Such an ecosystem would not only improve trial efficiency, but also provides a direct return for people with Parkinson syndromes supporting research by making them ‘findable’ and providing access to treatment innovation.

People living with neurodegenerative diseases and their caregivers should not have to wait for incremental change. Accelerating trial readiness and strengthening national coordination must therefore become a strategic priority for the German Parkinson research community, funding bodies, healthcare institutions, and policymakers alike.

### Limitations

This perspective reflects a single expert workshop and subsequent collaborative manuscript development, rather than a systematic evidence review or national performance audit. Participants’ observations may not represent all centres or stakeholder perspectives, and the proposals have not been evaluated prospectively. Selected policy reports, industry-association analyses and charity-reported funding provide context but differ in scope and reporting period. Their findings should not be interpreted as a Parkinson-specific international ranking. Patient and stakeholder consultation, feasibility assessment and prospective evaluation are needed to develop the proposals further.

## Data Availability

No datasets were generated or analysed during the current study.

## References

[CR1] Berg D, Postuma RB, Adler CH et al (2015) MDS research criteria for prodromal Parkinson’s disease. Mov Disord 30:1600–1611 10.1002/mds.26431 26474317

[CR2] Bernhardt AM, Longen S, Trossbach SV et al (2025) A quantitative Lewy-fold-specific alpha-synuclein seed amplification assay as a progression marker for Parkinson’s disease. Acta Neuropathol 149:20 10.1007/s00401-025-02853-y PMC11842418 39976789

[CR3] Bischof GN, Brendel M, Barthel H, Theis H, Barbe M, Bartenstein P, Claasen J, Danek A, Höglinger G, Levin J, Marek K, Neumaier B, Palleis C, Patt M, Rullmann M, Saur D, Schroeter ML, Seibyl J, Song M, Stephens A, Sabri O, Drzezga A, van Eimeren T, German Imaging Initiative for Tauopathies (2024) Improved Tau PET SUVR quantification in 4-repeat tau phenotypes with [18F]PI-2620. J Nucl Med 65(6):952–955 10.2967/jnumed.123.265930 PMC11149601 38575191

[CR4] Braak H, Del Tredici K, Rüb U et al (2003) Staging of brain pathology related to sporadic Parkinson’s disease. Neurobiol Aging 24:197–211 10.1016/s0197-4580(02)00065-9 12498954

[CR5] Brendel M, Barthel H, van Eimeren T et al (2020) Assessment of 18F-PI-2620 as a biomarker in progressive supranuclear palsy. JAMA Neurol 77:1408–1419 10.1001/jamaneurol.2020.2526 PMC7341407 33165511

[CR6] Brzenczek C, Klopfenstein Q, Hähnel T, Sapienza S, Klucken J, Fröhlich H, Glaab E, NCER-PD Consortium (2024) Integrating digital gait data with metabolomics and clinical data to predict outcomes in Parkinson’s disease. NPJ Digit Med 7(1):235 10.1038/s41746-024-01236-z PMC11379877 39242660

[CR7] Deuschl G, Schade-Brittinger C, Krack P et al (2006) A randomized trial of deep-brain stimulation for Parkinson’s disease. N Engl J Med 355:896–908 10.1056/NEJMoa060281 16943402

[CR8] van Eimeren T, Antonini A, Berg D, Bohnen N, Ceravolo R, Drzezga A, Höglinger GU, Higuchi M, Lehericy S, Lewis S, Monchi O, Nestor P, Ondrus M, Pavese N, Peralta MC, Piccini P, Pineda-Pardo JÁ, Rektorová I, Rodríguez-Oroz M, Rominger A, Seppi K, Stoessl AJ, Tessitore A, Thobois S, Kaasinen V, Wenning G, Siebner HR, Strafella AP, Rowe JB (2019) Neuroimaging biomarkers for clinical trials in atypical parkinsonian disorders: proposal for a Neuroimaging Biomarker Utility System. Alzheimers Dement (Amst) 11:301–309 10.1016/j.dadm.2019.01.011 PMC6446052 30984816

[CR9] Fomo M, Borga LG, Abel T, Santangelo PS, Riggare S, Klucken J, Paccoud I (2025) Empowering capabilities of people with chronic conditions supported by digital health technologies: scoping review. J Med Internet Res 27:e68458 10.2196/68458 PMC12254712 40577044

[CR10] Giehl K, Mutsaerts HJ, Aarts K, Barkhof F, Caspers S, Chetelat G, Colin ME, Düzel E, Frisoni GB, Ikram MA, Jovicich J, Morbelli S, Oertel W, Paret C, Perani D, Ritter P, Segura B, Wisse LEM, De Witte E, Cappa SF, van Eimeren T (2024) Sharing brain imaging data in the Open Science era: how and why? Lancet Digit Health 6(7):e526–e535 10.1016/S2589-7500(24)00069-4 38906618

[CR11] Höglinger GU, Respondek G, Stamelou M et al (2017) Clinical diagnosis of progressive supranuclear palsy: the Movement Disorder Society criteria. Mov Disord 32:853–864 10.1002/mds.26987 PMC5516529 28467028

[CR12] Höglinger GU, Berg D, Postuma RB et al (2024) A biological classification of Parkinson’s disease: the SynNeurGe research diagnostic criteria. Lancet Neurol 23:191–204 10.1016/S1474-4422(23)00404-0 38267191

[CR13] Koeglsperger T, Höllerhage M, Kassubek J, Woitalla D, Winkler J, Klucken J (2026) The future digital outpatient clinic for movement disorders: from technological innovation to ethical and systemic implementation-a position paper. J Neural Transm (Vienna). 10.1007/s00702-026-03167-3 PMC13743598 42133016

[CR14] Lill CM, Roehr JT, McQueen MB et al (2012) Comprehensive research synopsis and systematic meta-analyses in Parkinson’s disease genetics: the PDGene database. PLoS Genet 8:e1002548 10.1371/journal.pgen.1002548 PMC3305333 22438815

[CR15] Mikolaizak AS, Rochester L, Maetzler W, Sharrack B, Demeyer H, Mazzà C, Caulfield B, Garcia-Aymerich J, Vereijken B, Arnera V, Miller R, Piraino P, Ammour N, Gordon MF, Troosters T, Yarnall AJ, Alcock L, Gaßner H, Winkler J, Klucken J, Schlenstedt C, Watz H, Kirsten AM, Vogiatzis I, Chynkiamis N, Hume E, Megaritis D, Nieuwboer A, Ginis P, Buckley E, Brittain G, Comi G, Leocani L, Helbostad JL, Johnsen LG, Taraldsen K, Blain H, Driss V, Frei A, Puhan MA, Polhemus A, de Bosch M, Gimeno E, Hopkinson NS, Buttery SC, Hausdorff JM, Mirelman A, Evers J, Neatrour I, Singleton D, Schwickert L, Becker C (2022) Jansen CP; clinical validation study (WP4) on behalf of Mobilise-D consortium. Connecting real-world digital mobility assessment to clinical outcomes for regulatory and clinical endorsement-the Mobilise-D study protocol. PLoS ONE 17(10):e0269615 10.1371/journal.pone.0269615 PMC9536536 36201476

[CR16] Mollenhauer B, Locascio JJ, Schulz-Schaeffer W et al (2011) α-Synuclein and tau concentrations in cerebrospinal fluid of patients presenting with parkinsonism. Lancet Neurol 10:230–240 10.1016/S1474-4422(11)70014-X 21317042

[CR17] Mollenhauer B, Caspell-Garcia CJ, Coffey CS et al (2019) Longitudinal analyses of cerebrospinal fluid α-synuclein in prodromal and early Parkinson’s disease. Mov Disord 34:1354–1364 10.1002/mds.27806 PMC7098385 31361367

[CR18] Nalls MA, Pankratz N, Lill CM et al (2014) Large-scale meta-analysis of genome-wide association data identifies six new risk loci for Parkinson’s disease. Nat Genet 46:989–993 10.1038/ng.3043 PMC4146673 25064009

[CR19] Palleis C, Bernhardt AM, Weidinger E et al (2026) A biomarker-based classification of corticobasal syndrome. Mov Disord 41:129–142 10.1002/mds.70070 PMC12882055 41048081

[CR20] Parkinson Stiftung (2025) Sechs geförderte Leuchtturm-Projekte gestartet. 14 August 2025. https://www.parkinsonstiftung.de/artikel/leuchtturm-projektverbund-ist-gestartet/. Accessed 8 October 2026

[CR21] Parkinson’s UK (2026) We’re funding 3 new projects exploring ways of slowing down Parkinson’s. 2 February 2026. https://www.parkinsons.org.uk/news/2026/were-funding-3-new-projects-exploring-ways-slowing-down-parkinsons. Accessed 8 October 2026

[CR22] Peters J, Klammt S, Schindler C (2023) IITs – Investigator Initiated Trials: Kooperation von Akademia und Industrie. PM QM 25(3):10–19. 10.4126/FRL01-006472794

[CR23] Poewe WH, Rascol O, Quinn N et al (2007) Efficacy of pramipexole and transdermal rotigotine in advanced Parkinson’s disease: a double-blind, double-dummy, randomised controlled trial. Lancet Neurol 6:513–520 10.1016/S1474-4422(07)70108-4 17509486

[CR24] Postuma RB, Berg D, Stern M et al (2015) MDS clinical diagnostic criteria for Parkinson’s disease. Mov Disord 30:1591–1601 10.1002/mds.26424 26474316

[CR25] Raab R, Küderle A, Zakreuskaya A, Stern AD, Klucken J, Kaissis G, Rueckert D, Boll S, Eils R, Wagener H, Eskofier BM (2023) Federated electronic health records for the European Health Data Space. Lancet Digit Health 5(11):e840–e847 10.1016/S2589-7500(23)00156-5 37741765

[CR26] Respondek G, Höglinger GU (2021) DescribePSP and ProPSP: German multicenter networks for standardized prospective collection of clinical data, imaging data, and biomaterials of patients with progressive supranuclear palsy. Front Neurol 12:644064 10.3389/fneur.2021.644064 PMC8186498 34113306

[CR27] Riederer P, Strobel S, Nagatsu T, Watanabe H, Chen X, Löschmann PA, Sian-Hulsmann J, Jost WH, Müller T, Dijkstra JM, Monoranu CM (2025) Levodopa treatment: impacts and mechanisms throughout Parkinson’s disease progression. J Neural Transm (Vienna) 132(6):743–779 10.1007/s00702-025-02893-4 PMC12116664 40214767

[CR28] Saw RS, Haas S, Schmidt F, Ryazanov S, Leonov A, Bleher D, Grotegerd AK, Kuebler L, Roeben B, Schmidt F, Reimold M, Bonanno F, Ruf VC, Dahl B, Sandiego CM, Henry KE, Papadopoulos I, Schaller M, Kahle PJ, Levin J, Gasser T, Brockmann K, Reischl G, la Fougère C, Pichler BJ, Maurer A, Griesinger C, Giese A, Herfert K (2026) The PET tracer [(11)C]MODAG-005 targets alpha-synuclein aggregates in the brain. Sci Transl Med 18(851):eaec0813 10.1126/scitranslmed.aec0813 42202047

[CR29] Schuepbach WMM, Rau J, Knudsen K et al (2013) Neurostimulation for Parkinson’s disease with early motor complications. N Engl J Med 368:610–622 10.1056/NEJMoa1205158 23406026

[CR30] Simuni T, Chahine LM, Poston K, Brumm M, Buracchio T, Campbell M, Chowdhury S, Coffey C, Concha-Marambio L, Dam T, DiBiaso P, Foroud T, Frasier M, Gochanour C, Jennings D, Kieburtz K, Kopil CM, Merchant K, Mollenhauer B, Montine T, Nudelman K, Pagano G, Seibyl J, Sherer T, Singleton A, Stephenson D, Stern M, Soto C, Tanner CM, Tolosa E, Weintraub D, Xiao Y, Siderowf A, Dunn B, Marek K (2024) A biological definition of neuronal alpha-synuclein disease: towards an integrated staging system for research. Lancet Neurol 23(2):178–190 10.1016/S1474-4422(23)00405-2 38267190

[CR31] Smith KA, Ward T, Lambe S, Ostinelli EG, Blease C, Gant T, Gold SM, Holmes EA, Paccoud I, Vinnikova A, Klucken J, Uhlhaas PJ, Sanchez CG, Haining K, Böge K, Lahutina S, Tomelleri L, Ryan S, Torous J, Cipriani A (2025) Engagement and attrition in digital mental health: current challenges and potential solutions. NPJ Digit Med 8(1):398 10.1038/s41746-025-01778-w PMC12223045 40604240

[CR32] Spillantini MG, Schmidt ML, Lee VM, Trojanowski JQ, Jakes R, Goedert M (1997) Alpha-synuclein in Lewy bodies. Nature 388(6645):839–840 10.1038/42166 9278044

[CR33] Stamelou M, Respondek G, Giagkou N et al (2021) Evolving concepts in progressive supranuclear palsy and other 4-repeat tauopathies. Nat Rev Neurol 17:601–620 10.1038/s41582-021-00541-5 34426686

[CR34] Trenkwalder C, Kies B, Rudzinska M et al (2011) Rotigotine effects on early morning motor function and sleep in Parkinson’s disease: a double-blind, randomized, placebo-controlled study. Mov Disord 26:90–99 10.1002/mds.23441 PMC3072524 21322021

[CR35] Verband Forschender Arzneimittelhersteller e. V Deutschland verliert bei klinischen Studien weiter an Boden. ClinicalTrials.gov analysis of studies starting in 2023. https://www.vfa.de/de/arzneimittel-forschung/forschungsstandort-deutschland/klinische-studien-deutschland.html. Accessed 8 October 2026

[CR36] Wenning GK, Stankovic I, Vignatelli L et al (2022) The Movement Disorder Society criteria for the diagnosis of multiple system atrophy. Mov Disord 37:1131–1148 10.1002/mds.29005 PMC9321158 35445419

[CR37] Wissenschaftsrat (2018) Empfehlungen zu Klinischen Studien. Drs. 7301-18. Hannover, 19 October 2018. https://www.wissenschaftsrat.de/download/archiv/7301-18.html

[CR38] Zimprich A, Biskup S, Leitner P et al (2004) Mutations in LRRK2 cause autosomal-dominant parkinsonism with pleomorphic pathology. Neuron 44:601–607 10.1016/j.neuron.2004.11.005 15541309

